# Ten year follow-up of lung transplantations using initially rejected donor lungs after reconditioning using ex vivo lung perfusion

**DOI:** 10.1186/s13019-019-0948-1

**Published:** 2019-07-01

**Authors:** Haider Ghaidan, Mohammed Fakhro, Jesper Andreasson, Leif Pierre, Richard Ingemansson, Sandra Lindstedt

**Affiliations:** 1 Department of Cardiothoracic Surgery, Skåne University Hospital, Lund University, Lund, Sweden; 2 Wallenberg Center for Molecular Medicine, Lund University, 221 85 Lund, Sweden

**Keywords:** Lung transplantation, EVLP, Long-term follow up, Survival, Lung function

## Abstract

**Background:**

In 2006 and 2007 we performed double lung transplantation with marginal donor lungs assessed and reconditioned by Ex Vivo Lung Perfusion (EVLP), using a technique developed by Professor Stig Steen. Here we present a 10-year follow-up comparing the outcomes of lung transplantations performed at our clinic using EVLP lungs vs. conventional lungs.

**Method:**

Between 2006 and 2007, 21 patients (6 EVLP, 15 conventional) underwent double lung transplantation (LTx) with follow-up on May 2017 at Lund University Hospital, Sweden. Pulmonary function was measured at 3/6/12 months, and annually thereafter for a period of 10 years in addition to survival and freedom from chronic lung allograft dysfunction (CLAD) being analyzed.

**Results:**

Regarding Forced Expiratory Volume in 1 s (FEV1) and 6MWT at 3, 6, and 12 months and annually thereafter, no difference in median FEV1 nor 6MWT was found for EVLP-LTx vs. conventional-LTx (*p* > 0.05). No difference was shown in post-operative survival between EVLP-LTx vs. conventional LTx for patients with an overall survival up to 10-years (p > 0.05). The same pattern was shown in sub analyses for patients with a limited survival up to 1 and 5 years (p > 0.05).

**Conclusion:**

No superiority was found in conventional-LTx over EVLP-LTx, neither in long-term survival nor pulmonary function. No difference in CLAD-free survival was seen between the two groups. We believe that EVLP is a safe and effective method to use in LTx, greatly increasing the donor pool by improving marginal lungs and providing an objective assessment of the viability of marginal donor lungs.

## Introduction

Lung transplantation (LTx) is the golden standard for treating patients with irreversible end-stage pulmonary disease. A major challenge to LTx is the scarcity of donor organs resulting in deaths on the waiting list [1, 2]. Contrary to transplantation of other organs, only between 30% of potential donor organs are being utilized for LTx. This low proportion for acceptance of donor grafts is due to fact that the vast majority of grafts do not meet the criteria for transplantation [3, 4]. In 2006 we transplanted the first six patients with double LTX (DLTx) performed with donor lungs reconditioned ex vivo. The lungs were rejected for transplantation by the Scandia-transplant, Euro-transplant, and UK- transplant organizations due to arterial oxygen pressure less than 40 kPa. The donor lungs were reconditioned ex vivo in an extracorporeal membrane oxygenation circuit with STEEN solution mixed with erythrocytes. Ex vivo lung perfusion (EVLP) is today used at many lung transplant centers all over the world and is considered to be the golden standard in assessing and reconditioning marginal donor lungs for transplantation [5–9]. We have earlier reported the promising early outcome of these first six patients with a three-month survival at 100% while four of these patients showed no signs of Chronic Lung Allograft Dysfunction (CLAD) two years post-transplant [6] .

Long-term outcome including survival, pulmonary function, and occurrence of CLAD is to some extent unknown regarding EVLP as is whether there is any disadvantage compared to conventional LTx. This retrospective cohort study reviews the 10-year follow-up of the first six transplanted recipients. The study also compares EVLP DLTx to conventional DLTx performed at our center during the same time period.

## Patients and methods

### Data source

Between June 2006 and April 2007, 21 patients underwent LTx at Skåne University Hospital in Lund. Out of these, all were double-lung transplanted (DLTx). The median age for these patients was 52 years with a range of 22–66 years. In terms of gender, 9 were males and 12 females. The major indications for an LTx were chronic obstructive pulmonary disease (COPD) (*n* = 8), cystic fibrosis (CF) (n = 8), α1-antitrypsin deficiency (AAT1) (*n* = 1), pulmonary fibrosis (PF) (*n* = 2), pulmonary hypertension (PH) (n = 1), and lymphangioleiomyomatosis (LAM) (n = 1). With a total of 21 LTx, 6 were transplanted using EVLP, and 15 were transplanted using conventional LTx. This study has been performed in accordance with the Declaration of Helsinki and is approved by the Ethics Committee at Lund University with reference number 2016/638.

### Marginal donors

The data from the marginal donors has been reported and described in detail from our previous publication [7]. Originally, nine donor lungs were investigated. To undergo reconditioning, the same donor criteria were met for ordinary donor lungs as for marginal lungs with the addition that we accepted lower partial pressure in arterial blood (PaO2). All lungs were turned down due to non-acceptable oxygenation capacity. The beforehand decided criteria to be accepted for transplantation after reconditioning in EVLP was that the PaO2 on fraction of inspired oxygen (FiO2) = 1 should be 50 kPa or higher. Median donor age with range was 59 (34–63) and median weight (kg) with range was 76 (55–94). All but one donor was CMV positive. Three of the donors had a known smoking history. All the six donor grafts had a median PaO_2_ (KPa) before harvesting (FIO_2_ = 1.0) of 21.1 with a range of 11.5 to 28.7.

### EVLP setup

Our EVLP setup has been described in detail regarding our methodology in our previous studies [5, 7, 10].

### Spirometry (FEV1)

Patients followed a planned clinical regime and were reviewed at regular intervals (3, 6, and 12 months, and annually thereafter for a period of 10 years). Spirometry were performed at each follow-up, assessing the patient’s FEV1 (liters).

### 6-min walking test

A 6MWT was performed at each follow-up with a regime similar to spirometry (3, 6, and 12 months, and annually thereafter). Assessing the patient’s expected work percentage determined on walking distance (meters), age (years), height (cm), and weight (kg).

### Chronic lung allograft dysfunction (CLAD)

CLAD is a term that was introduced at first in 2010 [11, 12]. CLAD is principally caused from chronic rejection, usually resulting into one of following phenotypes: bronchiolitis obliterans (OB), neutrophilic reversible allograft dysfunction (NRAD), and restrictive allograft syndrome (RAS). Each of these conditions are usually presents with either airway obstruction or restriction and are mostly unresponsive to alterations in immunosuppression. According to the International Society for Heart and Lung Transplantation (ISHLT) guidelines, BOS, a major component of CLAD, is defined as more than 20% decline in FEV1 from the highest obtained baseline [12, 13], and is characterized by perivascular and interstitial mononuclear cell infiltrates or chronic rejection characterized by dense scarring and eosinophilic infiltrates. If rapid deterioration of pulmonary function was detected as a sign of CLAD, bronchoscopies with TBB were conducted and anti-rejection treatment was initiated with pulsed metylprednisolon often together with tacrolimus or everolimus as a replacement for cyclosporine.

### Statistical methods

Data are presented as mean with standard deviation (SD), median with range, or frequency with percentage. Shapiro-Wilks test was used to determine which variables were normally distributed/parametric (mean, SD) vs. non-normally distributed/non-parametric (median, range). Independent (unpaired) student’s t-test was conducted for normally distributed continuous variables while Mann-Whitney U (Wilcoxon rank sum) test was used for non-normally distributed continuous data. Chi-square test or Fisher’s exact test were chosen for analysis of categorical variables. For survival analysis, the end-point used was death or Re-LTx. For freedom from CLAD analysis, the endpoint used was occurrence of CLAD until death/Re-LTx/follow-up. Cox regression in accordance with Cox proportional hazards model was performed for univariable survival analysis and freedom from CLAD analysis. Survival/freedom from CLAD-estimates were displayed in accordance with Kaplan-Meier with log-rank test to detect significance between survival/freedom from CLAD curves. A *p*-value < 0.05 was considered statistically significant. Statistical analyses were performed using SPSS Version 24.0 (IBM Corp., Armonk, NY, USA).

## Results

### Recipient characteristics

Baseline and clinical characteristics of recipients between EVLP-LTx and conventional-LTx are shown in Table 1. No significant difference between EVLP-LTx and conventional-LTx were shown regarding pulmonary function (FVC, FEV1, 6MWT), liver/kidney-status (AST, ALT, creatinine), and pre-operative life support (ECMO or mechanical ventilation) (*p* > 0.05).

**Table 1:** Recipient baseline and clinical characteristics of ex vivo lung perfusion (EVLP) lung transplantations (LTX) and conventional LTx

Variables	EVLP-LTx (*n* = 6)	Conventional-LTx (*n* = 15)	*p*-value
Weight (kg)	70.7 ± 19.3	59. 1 ± 7.9	0.060
Height (cm)	170.8 ± 11.8	169.9 ± 10.1	0.862
BMI	24.0 ± 5.3	20.5 ± 3.5	0.088
Male	3 (50%)	6 (40%)	0.523
Age (years)	54.1 ± 10.4	42.6 ± 14.8	0.100
Waiting list (days)	49.0 (7–174)	44 (4–389)	0.785
*Pre-op Life support*			
Mechanical ventilation	0 (0.00%)	1 (6.66%)	0.714
ECMO	0 (0.00%)	1 (6.66%)	0.750
*Major indication*			0.407
COPD	3 (50.00%)	5 (33.33%)	
AAT1	1 (16.66%)	0 (0.00%)	
PH	0 (0.00%)	1 (6.66%)	
CF	1 (16.66%)	7 (46.66%)	
PF	1 (16.66%)	1 (6.66%)	
LAM	0 (0.00%)	1 (6.66%)	
*Lab values*			
FVC (liters)	2.0 ± 0.4	1.0 ± 0.6	0.540
FEV1 (liters)	0.8 ± 10.4	54.1 ± 10.4	0.516
6MWT (%)	39.6 ± 21.4	45.9 ± 25.1	0.600
P-ALT (μkat/L)	0.41 ± 0.15	0.32 ± 10.4	0.181
P-AST (μkat/L)	0.46 ± 0.12	0.41 ± 0.11	0.443
P-creatinine (μmol/L)	64.4 ± 11.5	54.1 ± 15.8	0. 216
*Tx-type*			
SLTx	0 (0%)	0 (0%)	
DLTx	6 (100%)	15 (100%)	
HLTx	0 (0%)	0 (0%)	
Re-LTx	0 (0%)	0 (0%)	

Data are mean (SD), number (%), or median (range). The numbers are based on patients with available data. COPD, chronic obstructive pulmonary disease; AAT1, Alpha 1-antitrypsin deficiency; PH, pulmonary hypertension; CF, cystic fibrosis; PF, pulmonary fibrosis; LAM, Lymphangioleiomyomatosis; BMI, body-mass index; FVC, forced volume vital capacity; FEV1, forced volume expiratory capacity 1 s; 6MWT, 6-min walking test; AST, aspartate transaminase; ALT, alanine transaminase; SLTx, single-lung transplantation; DLTx, double-lung transplantation; HLTx, heart-lung transplantation; Re-LTx, re-lungtransplantation; ECMO, extracorporeal membrane oxygenation

### Mortality

Cause of death during follow-up stratified between EVLP-LTx and conventional-LTx is illustrated in Table 2. No difference was found in cause of death (rejection, infection, malignancy, or “miscellaneous”) between EVLP-LTx vs. conventional-LTx (p > 0.05).

**Table 2:** Cause of death after transplantation between ex vivo lung perfusion (EVLP) lung transplantations (LTX) and conventional LTx

	EVLP-LTx (n = 6)	Conventional-LTx (n = 15)	*p*-value
Cause of death			0.406
*Total number of deaths*	3	6	
Death from Organ Rejection	2 (66.66%)	2 (33.33%)	
Death from Infection	0 (0.00%)	2 (33.33%)	
Death from Malignancy	0 (0.00%)	1 (16.66%)	
Death from Miscellaneous	1 (33.33%)	1 (16.66%)	

The group “Death from Miscellaneous” includes patients with mortality caused by myocardial and cerebral ischaemia, multiple organ failure such as renal and liver failure, as well as other causes related to the patient’s age and individual health status

### Survival/freedom from CLAD estimates

**Fig. 1:**
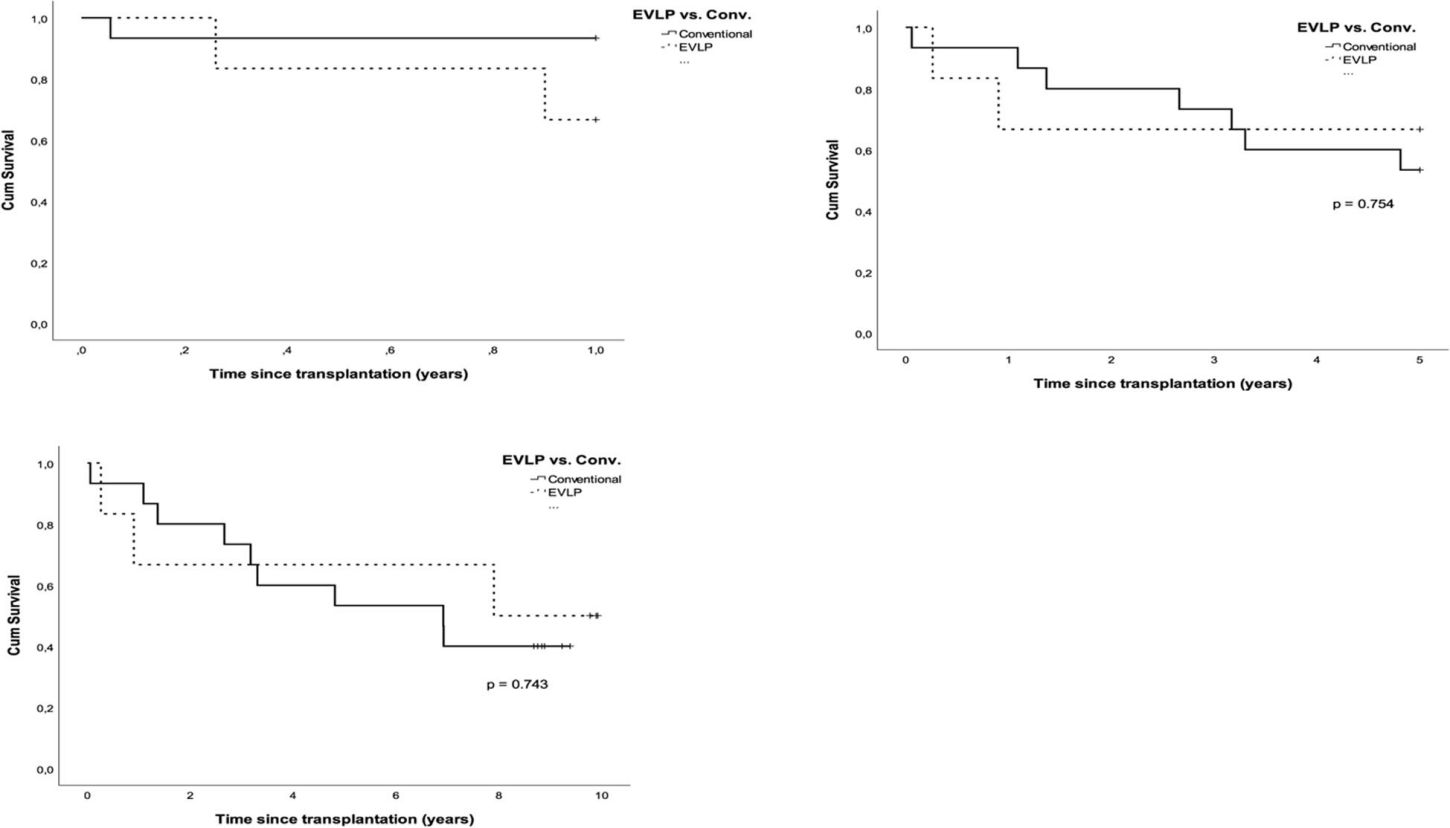
The upper right Kaplan-Meier figure illustrates post-transplant survival for ex vivo lung perfusion (EVLP) lung transplantation (LTx) vs. conventional LTx for recipients transplanted between 2006 and 2007 with a limited survival up to 1 year (*p* > 0.05) while the upper left figure displays recipients with a limited survival up to 5 years. The bottom figure displays overall post-transplant survival in the 10-year experience for LTx-recipients (EVLP-LTx and conventional LTx) (*p* > 0.05)

Cumulative survival rate estimates for EVLP-LTx and conventional-LTx at 1, 3, 5, and 10 years are illustrated in terms of percentage with an upper/lower 95% confidence interval (CI) (Fig. 1). For the entire cohort, EVLP-LTx showed 1-, 5-, and 7-year survival rates of 67% (CI 48–86), 67% (CI 48–86), and 50% (CI 30–70), respectively, compared to patients with conventional-LTx with 1-, 3-, 5-, and 7-year survival rates of 93% (CI 87–99), 73% (CI 62–85), 53% (CI 40–66), and 40% (CI 27–53), respectively (*p* > 0.05). In addition, survival at 1 year and 5 years showed no significant difference between EVLP-LTx and conventional-LTx (p > 0.05).

**Fig. 2:**
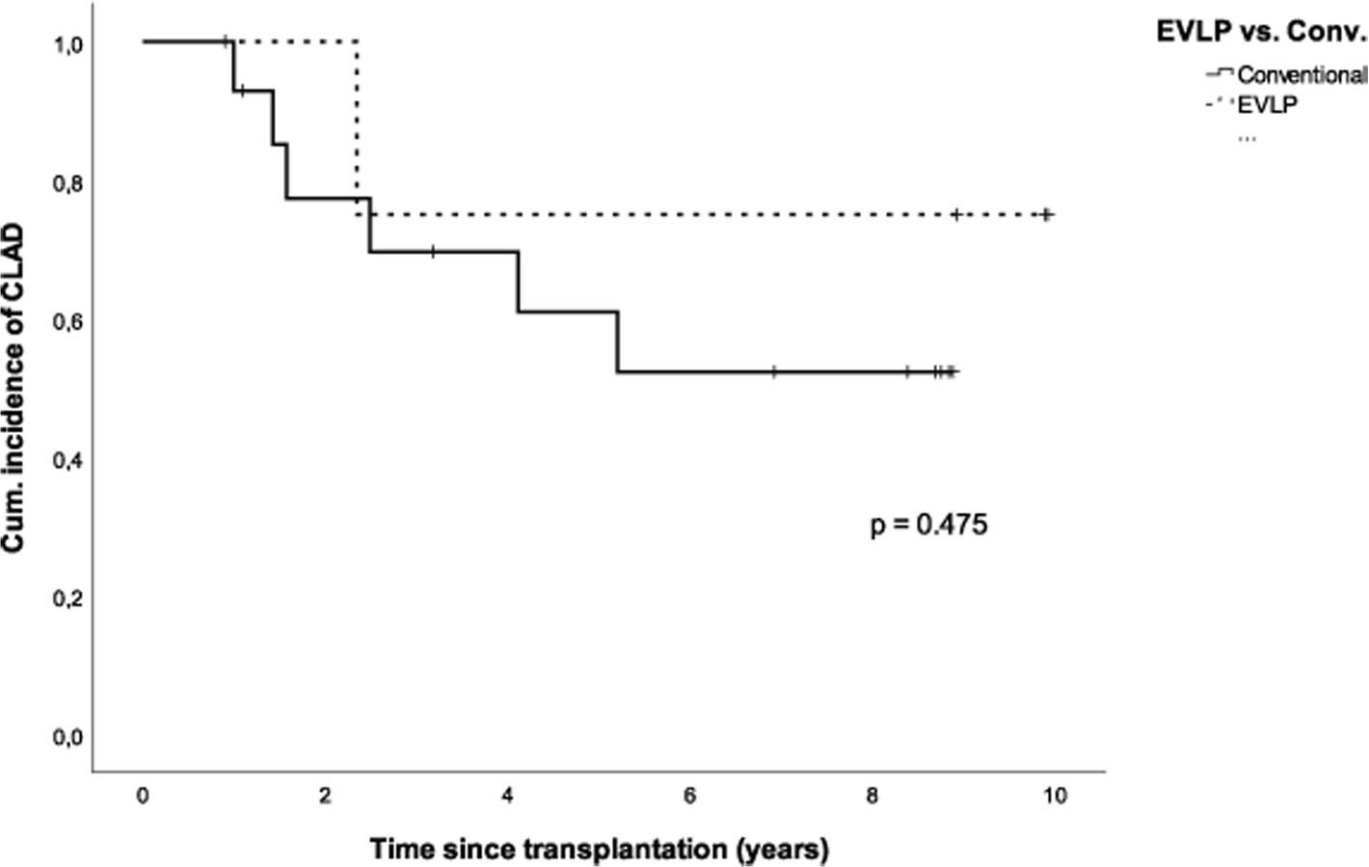
Kaplan-Meier figure displaying freedom from CLAD for ex vivo lung perfusion (EVLP) lung transplantation (LTx) vs. conventional LTx for recipients transplanted between 2006 and 2007 until follow-up or death/Re-LTx (*p* < 0.05)

Freedom from CLAD estimates are shown in Fig. 2. Conventional-LTx showed freedom from CLAD rates at 1, 3, 5, and 7 years at 93% (CI 86–100), 70% (CI 45–94), 61% (CI 34–88), and 52% (CI 24–80), respectively, compared to patients with EVLP-LTx at 1- and 3-year rates of 100 and 75% (CI 53–97) (*p* > 0.05).

### Cox regression

The Cox proportional hazards model (univariable) evaluating EVLP-LTx vs. conventional-LTx in addition to survival and freedom from CLAD is shown in Table 3. No significant difference was found between EVLP-LTx and conventional-LTx in overall survival of patients or in patients with a limited survival up to 1 and 5 years (*p* > 0.05). The same trend was found in the Cox regression analysis regarding freedom from CLAD regarding EVLP-LTx as opposed to conventional-LTx (*p* > 0.05).

**Table 3:** Cox proportional hazards model (univariable) ex vivo lung perfusion (EVLP) lung transplantations (LTX) and conventional LTx, evaluating survival and freedom from chronic allograft dysfunction (CLAD)

	HR	95% CI	*p*-value
Overall survival			
EVLP	1. 245	0. 335–4. 633	0. 744
5-year limited survival			
EVLP	1. 286	0. 266–6. 206	0. 754
1-year limited survival			
EVLP	0. 197	0. 018–2. 175	0. 185
Freedom from CLAD			
EVLP	0. 470	0. 057–3. 917	0. 486

*CI* Confidence interval, *HR* Hazard ratio

### Median FEV1 and 6MWT

**Fig. 3:**
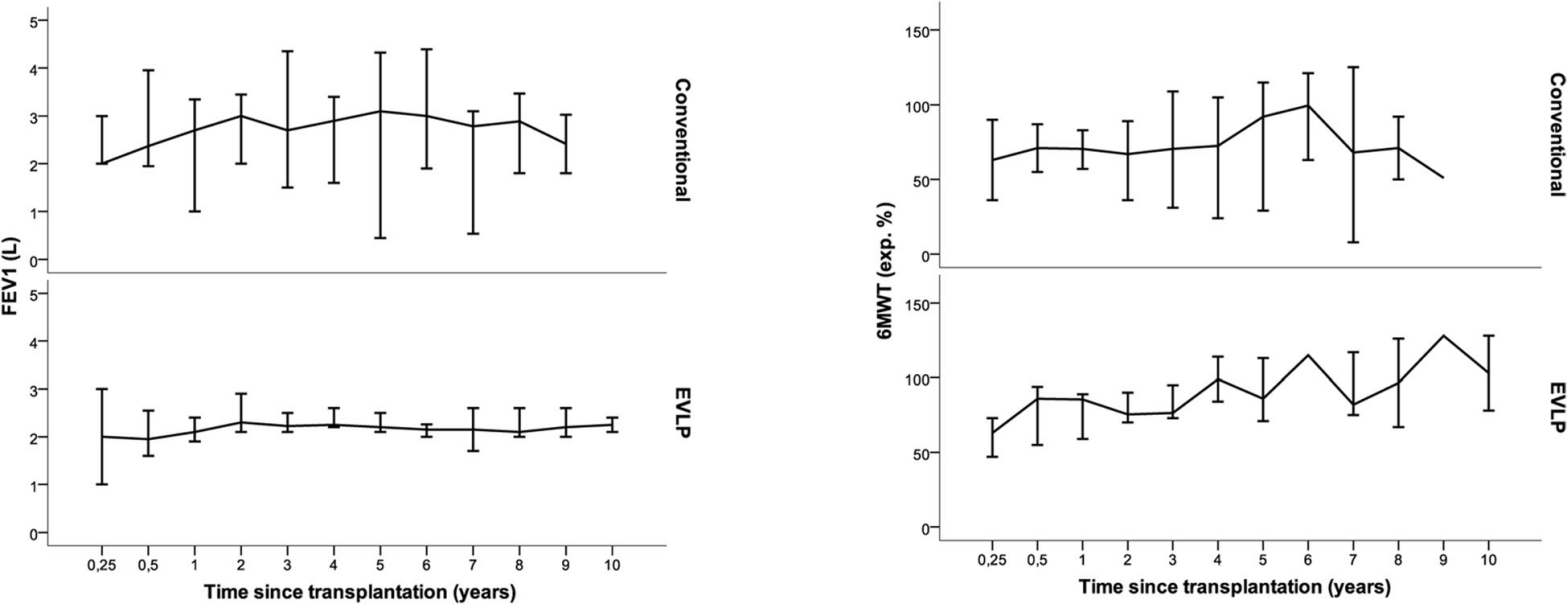
Median pulmonary function with 95% confidence interval is shown over time (years) after lung transplantation (LTx). Median forced expiratory volume in 1 s (FEV1) in liters is displayed to the right while 6-min walking test (6MWT) in expected work percentage is shown to the left for Ex vivo Lung Perfusion (EVLP) -LTx and conventional-LTx, respectively

Median pulmonary function with 95% CI over time regarding FEV1 in liters (L) and 6MWT in expected work percentage (%) is shown in Fig. 3. FEV1 for EVLP-LTx at 1, 5, and 7 years were 2.1 L (1.9–2.2), 2.2 L (2.1–2.5), and 2.1 (1.7–2.6) while conventional LTx showed at the same time intervals 2.6 L (1.0–3.3), 3.0 L (0.4–4.2), and 2.9 (0.5–3.1), respectively (*p* > 0.05). For the 6MWT, EVLP-LTx at 1, 5, and 7 years were 83% (57–87), 84% (70–112), and 79% (74–119), while conventional LTx was 71% (55–79), 88% (28–115), and 69% (10–123), respectively, at the same time intervals (p > 0.05).

## Discussion

A new era is at hand in the field of LTx due to the immense potential of EVLP. The number of patients who are in need of a LTx surpasses the amount of available grafts. Using cellular and normothermic perfusion in combination with ventilation, EVLP improves marginal lungs and allows for an objective assessment of their viability. By increasing the availability of donor organs for transplantation, EVLP is being established as the new cornerstone in LTx. The very first LTx evaluated ex vivo was performed in 2001 by Steen et al. at our center in Lund, Sweden, from a non-heart beating donor [14]. A few years later we were again the first team to successfully perform a series of LTx by using grafts that did not meet standard transplantation criteria (i.e. marginal donor lungs) [7], with significant lower PaO_2_ levels. In the present study, we present our 10-year experience regarding the short- and long-term outcome of the first six patients that underwent LTx using marginal donor lungs evaluated using EVLP at our center. We also compare the outcome with conventional LTx performed at our clinic during the same time period. None of the marginal donor lungs would have normally been used for LTx if not for EVLP at our center. The marginal donor lungs used had been rejected by national and international transplantation centers due to low PaO_2_. The Lund EVLP protocol include priming the system with Steen solution and red blood cells with a target hematocrit at 14%, the pressure in the left atrium is kept at 0 mmHg by leaving the atrium open. The pulmonary artery pressure is kept under 20 mmHg to avoid pulmonary edema. During the evaluation the cardiac output target flow is 100%. Our EVLP protocols have been described in detail in our previous publications [7, 10].

Long term follow-up after EVLP has been limited by the recent introduction of the technique in clinical LTx. We have previously reported our short-term EVLP experience with a 100% survival rate at 30 days, with no significant differences shown between EVLP and standard LTx regarding time on mechanical ventilation, ICU stay, and overall hospital stay [6].

Up to 12 months after transplantation, Fildes et al. [15] reported no difference in mortality or number of infections between EVLP-LTx and conventional LTx. Wallinder et al. having one of the longest follow-ups with their 4-year experience, were able to demonstrate that conventional LTx lungs showed no superiority over EVLP-LTx in terms of survival and post-operative complications [16]. Our 10-year experience, the longest clinical EVLP follow-up to date, detected no significant difference between EVLP-LTx vs. conventional-LTx. The same pattern emerged in our sub analyses concerning patients with a limited survival up to 1 and 5 years. These findings, along with the fact that all EVLP donor lungs were initially rejected for LTx, are highly encouraging as it may allow for the expansion of the donor pool with grafts from marginal donor lungs. An important report that has studied EVLP for marginal donors is the NOVEL Lung trial, an FDA-mandated multicenter clinical trial that included 31 patients who received EVLP lungs [17]. Early outcomes in this trial regarding ICU/hospital stay, time on mechanical ventilation, primary graft dysfunction, and 30-day mortality showed great promise as they were similar to the control group consisting of 31 conventional LTx. It has been hypothesized in previous studies that EVLP provides its protective effect on the graft by interrupting possible cold storage injury and resuming the lungs to its proper physiological metabolic state. In addition, it decreases the microbial load from the donor, thereby protecting the immunosuppressed LTx-recipient from infection [18, 19].

In the present study no significant difference was found in freedom from CLAD between EVLP-LTx and conventional-LTx. This finding is in accordance with previous studies showing similar outcomes between the two groups concerning freedom from CLAD up to five years after LTx [16, 20]. Pulmonary function was followed up in our clinical program by FEV1 and 6MWT at 3, 6, and 12 months and annually thereafter for a period of ten years. Interestingly, no superiority was recognized regarding conventional-LTx over EVLP-LTx. FEV1 and 6MWT are well-known clinical tools that are non-invasive and provide excellent data on the clinical status of the recipient after transplantation [21]. To the authors knowledge, this is the first time that long-term outcome regarding pulmonary function has been investigated between the two groups. This finding is highly encouraging as it provides further evidence that EVLP is a reliable instrument in LTx for increasing the donor pool. EVLP has been suggested as a platform for administering medical agents and thus improving patient outcome. It has been shown that inhalation of CO prevents ischemia-reperfusion injury in animal models. It has also been shown that administration of nitroglycerin, selective adenosine 2A agonists, and dibutyryl cyclic adeonosine monophosphate in Steen solution lowers the rate of histological graft injury, lowers the levels of inflammatory cytokines, and keeps the microvascular structure of the graft unharmed compared to conventional LTx [22, 23]. It has also been reported that decreasing levels of thyroid stimulating hormone and desmopressin in brain-dead donors might have a role to play in LTx, where administration of such hormone replacements are made possible through EVLP and could improve graft function [24]. EVLP may also play a role in donation after circulatory death (DCD) especially in uncontrolled DCD donors when lung function is often unknown. Interestingly it has been reported that DCD-lungs that underwent EVLP showed better outcome regarding length of hospital stay and time on mechanical ventilation [25].

As EVLP allows for the extension of preservation time, it has the potential to open up possibilities for safer procedures by enabling more daytime surgery [26]. Extra preservation time could also reduce geographical limitations for recipients and donors, thus increasing both the safety and number of LTx [8].

EVLP might also serve as an important bridge in the future taking ex vivo lung bioengineering approaches to create functional, transplantable grafts into the clinic. Ex vivo lung bioengineering has the potential to overcome the challenges of organ donor shortages and prevent allograft immune rejection [27].

### Limitations

This study is not without limitations. Only a small number of cases were followed up in our single center study. While a randomized clinical study of EVLP is ideal, it is not currently feasible due to ethical disagreement in the clinic about transplanting such grafts. It is difficult, therefore, to provide a true comparison between EVLP and conventional LTx. A larger dataset and equally large groups could give a more powerful analysis and limit the occurrence of type II errors.

## Conclusions

On average 40% of brain dead donor lungs do not meet the criteria for LTx and are therefore not accepted for LTx. A considerable number of these organs may have been utilized in LTx through EVLP, which provides a method to evaluate and improve marginal donor lungs. According to the findings of our 10-year follow-up, the longest clinical follow-up to date, no differences were found between conventional LTx and EVLP regarding survival, pulmonary function, or incidence of CLAD.

## Data Availability

Please contact author for data requests.

## Abbreviations

6MWT: 6 min walking test
CLAD: Chronic lung allograft dysfunction
DCD: Donation after circulatory death
DLTx: Double lung transplantation
EVLP: Ex vivo lung perfusion
FVC: Forces vital capacity
ISHLT: International society of heart and lung transplantation
LTx: Lung transplantation
Re-LTx: Redo lung transplantation
SLTx: Single lung transplantation
